# Pet Pyometra: Correlating Bacteria Pathogenicity to Endometrial Histological Changes

**DOI:** 10.3390/pathogens10070833

**Published:** 2021-07-02

**Authors:** Cassiane Elisabete Lopes, Silvia De Carli, Camila Imperico Riboldi, Cíntia De Lorenzo, Welden Panziera, David Driemeier, Franciele Maboni Siqueira

**Affiliations:** 1 Laboratory of Veterinary Bacteriology, Veterinary School, Federal University of Rio Grande do Sul (UFRGS), Porto Alegre 91540-000, Brazil; cassianeelisabete@gmail.com (C.E.L.); silvia.decarli93@gmail.com (S.D.C.); camilaimperico@hotmail.com (C.I.R.); 2 Laboratory of Veterinary Pathology, Veterinary School, Federal University of Rio Grande do Sul (UFRGS), Porto Alegre 91540-000, Brazil; cintiadelorenzobr@gmail.com (C.D.L.); weldenpanziera@yahoo.com.br (W.P.); ddriemeier@gmail.com (D.D.)

**Keywords:** uterus infection, companion animals, bacteria isolation, virulence genes, histopathology, EnPEC

## Abstract

Pyometra is a life-threatening infectious disease that frequently affects bitches and queens. Although histopathological patterns of pyometra have been extensively explored, the microbiological aspects, such as bacteria pathogenicity, have not been correlated to microscopy endometrial lesions so far. In this study, these two pathological aspects of pyometra were analysed and correlated. Uterus fragments and intrauterine content samples were collected from pets diagnosed with pyometra (30) and submitted to histopathology analysis and bacterial culture, respectively. The degree of endometrial histopathological lesions in pyometra cases were classified as mild, moderate and severe. Thirty different bacteria isolates were identified from intrauterine content culture. *Escherichia coli* (*E. coli*) was pure isolated in 57.7% and highly related to severe endometrial lesions. Immunohistochemistry assay revealed the adhesion and invasion of this bacteria agent to the injured endometrium. Virulence aspects of these *E. coli* strains were explored, demonstrating biofilm formation ability and a set of virulence genes in most isolates. These results support the adaptive genetic and phenotypic advantages of *E. coli* for uterus infection, and justify the high frequency of this agent involved in pyometra cases.

## 1. Introduction

Pyometra is a suppurative infection with the accumulation of purulent exudate within the uterine lumen. In companion animals (bitches and queens), pyometra is usually acute and life-threatening, requiring surgical treatment (ovariohysterectomy). The pathogenesis of pyometra remains unknown; however, hormonal conditions and bacteria virulence contributes to endometrial changes [1]. Cystic endometrial hyperplasia–pyometra complex is the condition proposed by pathologists to describe the series of lesions observed microscopically in pet pyometra cases [2]. However, the requirement of endometrial hyperplasia to the development of pyometra is still controversial [3]. 

In this context, little is known about the contribution of bacteria pathogenicity to the severity of the lesions. *Escherichia coli* (*E. coli*) is the most frequent bacteria isolated from pyometra in companion animals and is associated to severe clinical cases [4,5]. Other bacteria genus, even lowest frequency, also have been involved in pyometra infections, such *Staphylococcus* spp., *Streptococcus* spp., *Pseudomonas* spp., *Proteus* spp., *Enterobacter* spp., *Nocardia* spp., *Pasteurella* spp., and *Klebsiella* spp. [6]. 

Although both microorganisms and pathological lesions involved in pyometra are well established, no relationship between these aspects have been traced so far. Therefore, the main proposal of this study was to determine correlations among different degrees of endometrial histopathological lesions and bacteria isolation from intrauterine purulent content from bitches and queens with suspected pyometra infections. Additionally, phenotypic and genotypic assays were performed with the most frequent bacteria to access their pathogenic profile.

## 2. Results

### 2.1. Bacterial Isolates from Intrauterine Content

After 48 h of incubation, 86.7% (26/30) of the intrauterine content from pyometra cases showed bacteria growth on blood agar in aerobiosis atmosphere. Growth on MacConkey agar was observed in 70% (21/30) of the inoculations. Exclusive growth under neither anaerobiosis nor microaerophilia was detected. From the 26 culture-positive samples, 30 different bacteria isolates were identified at the secure specie or genus level (Table 1) with a median MALDI-TOF log-score of 2.38 (IQR: 2.25–2.44). A total of 15 pure growths on sheep blood agar and MacConkey agar of *E. coli* were obtained from the pyometra cases (57.7%). The other bacteria isolated as pure culture were *Enterococcus* sp. (15.4%), *Streptococcus* sp. (11.5%), and *Pseudomonas aeruginosa* (*P. aeruginosa*) (3.8%). In three culture-positive samples (11.5%) association between two genera was observed (Table 1). Interestingly, in all cases with *Staphylococcus* spp. Isolation, there was co-infection with other bacteria, such as *E. coli*, Bacillus sp. or *Streptococcus* sp. (Table 1). Finally, uterus mucus from the control group, which comprised the eight females without pyometra infection, had no bacterial isolation (*p* < 0.05).

### 2.2. Classification of the Endometrial Histopathological Lesions from Pet Pyometra

**Figure 1:**
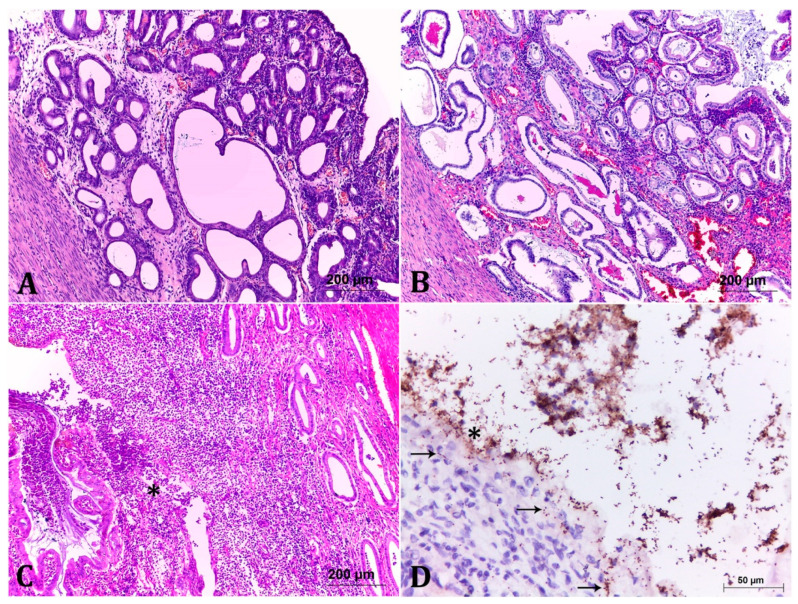
Histological analysis of the degrees of endometrial lesions in cases of pyometra. (**A**) Mild lesion. There is discrete hyperplasia of endometrial glands, with occasional ectasia. In addition, there are foci of discrete inflammatory infiltrate in the interstices of the glands. HE, 10×. (**B**) Moderate lesion. It shows more pronounced hyperplastic and ectatic lesions when compared to Figure 1A, associated to moderate interstitial inflammatory infiltrate of lymphocytes, plasma cells, macrophages and neutrophils. The endometrial and glandular epithelium are vacuolated and occasionally contain several layers. Some glands are filled with basophilic or amorphous eosinophilic material. HE, 10×. (**C**) Severe lesion. There is marked mixed inflammatory infiltrate (lymphocytes, plasma cells, macrophages and neutrophils) associated with marked endometrial gland necrosis (asterisk). Some glands are lumen filled with cellular debris and the remaining epithelium is vacuolated and often infiltrated by inflammatory cells. HE, 10×. (**D**) Anti-*E. coli* immunohistochemistry of the strain LBV_029/18. Accentuated and diffuse immunostaining of multiple coccobacilli adhered to the lining epithelium and in the medium to the content in the uterine lumen. Note that there are often bacteria inside the epithelial cells (arrow) and in the interstice of the endometrium (arrow) illustrating the invasive profile, 40×.

To establish a correlation between bacterial isolation and endometrial pathological changes, we evaluated the same 30 pyometra cases histopathologically. Briefly, three groups were classified according to the severity degrees of the histopathological findings. No histopathological observations in the negative control group were observed (Table 1; *p* < 0.05). All ovaries of the analysed animals presented corpus luteum. Mixed bacterial cultures (co-infections) were observed in three cases, each one with a different severity degree: mild, moderate, and severe (Table 1). Lesions classified as mild lesions, corresponding to 13.3% (4/30) (Table 1), were characterized by restricted alterations to the endometrium, mainly by focal areas of discrete hyperplasia in the endometrial glands, including occasional ectasia. In lamina propria and submucosa, a discrete inflammatory infiltrate composed of lymphocytes, plasma cells, macrophages, and neutrophils were visualized (Figure 1A). Bacteria involved in mild lesions were *Bacillus* sp. in association to *Staphylococcus* sp. (1/4), and *Enterococcus* sp. (1/4) (Table 1). The two remaining cases (2/4; 50%) classified as mild lesions were negative to microbiological culture. No statistical differences were observed among bacteria species isolated in mild lesions in the one-way ANOVA test.

Uterine lesions classified as moderate lesions (9/30; 30% of cases) presented more pronounced degrees of endometrial hyperplasia and ectasia, associated to moderate inflammatory infiltrate. In some areas, the endometrial and glandular epithelium were hyperplastic, vacuolated, with foci of necrosis, in addition to neutrophil invasion and often contained granular basophilic material (Figure 1B). In some cases, focal inflammatory infiltrate in the myometrium was observed (Figure 1B). Bacteria involved in the described lesion degrees were mainly *E. coli* alone (3/9) and in one case (1/9) in association with *Staphylcoccus pseudintermedius* (*S. pseudintermedius*), followed by *Enterococcus* sp. (2/9), *Streptococcus* sp. (2/9), and *E. coli* in association with *S. pseudintermedius* (1/9) (Table 1). As in the previous group, no statistical difference was obtained among the bacteria species isolated in moderate lesions at the one-way ANOVA test.

Seventeen samples (56.7%) corresponded to the group with severe lesions. The changes were similar to those described in the previous group, with a higher degree of intensity. Additionally, there were areas of marked endometrial necrosis, associated with pronounced mixed inflammatory infiltrate, which also occluded the uterine lumen, in some cases with lack of endometrial architecture (Figure 1C). *E. coli* was the predominant pathogen isolated from the severe cases with 70.6% (12/17). Other bacteria present in severe lesions were: *Enterococcus* sp., *P. aeruginosa*, *Streptococcus* sp., and *S. pseudintermedius* in association with *Streptococcus* sp. The one-way ANOVA test demonstrates statistical differences among the species isolated in the severe lesion cases (*p* < 0.05). Tukey’s test indicates that *E. coli* is the most frequent bacteria in these cases (*p* < 0.05). 

In order to further correlate the *E. coli* isolation with one or more endometrial histopathological lesion degrees, the Cochran–Armitage test was performed and demonstrated a positive correlation between *E. coli* isolation in intrauterine content and the severe lesion classification on histopathological analysis. No more correlations were statistically significant in our analysis. As *E. coli* was the most common bacteria identified and was highly related to severe lesions, we better characterized *E. coli* isolates, in order to establish a possible relationship between the virulence potential of the isolated strains and uterine lesions, especially the severe ones.

### 2.3. Immunohistochemistry (IHC) Showed Important Aspects about E. coli Intrauterine Infection

In order to show the pathogenic ability of the *E. coli* strains, two samples of uterine tissues classified as severe lesion (LBV_029/18 and LBV_037/18) with pure culture of *E. coli* were marked to IHC staining with antibodies anti-*E. coli*. Tissue staining demonstrated multiple coccobacilli adhered to the lining epithelium, the invasion of epithelial cells, in addition to necrosis of the tissue (Figure 1D).

### 2.4. Extracellular Matrix Components Expression and In Vitro Biofilm Formation Were Widespread among the E. coli Strains

The expression of curli and cellulose influences the formation of biofilms; therefore, phenotypic expression of these extracellular matrix components was analysed, in addition to the in vitro biofilm formation ability of the *E. coli* strains. Twelve *E. coli* strains (80%) produced both curli and cellulose, corresponding to the rdar morphotype, while three strains produced neither curli nor cellulose, representing the saw morphotype (Table 2). Interestingly, the three strains with saw morphotype were able to produce biofilm in the tested conditions. In contrast, one strain presenting the rdar morphotype was not able to form biofilm in the in vitro conditions.

**Figure 2:**
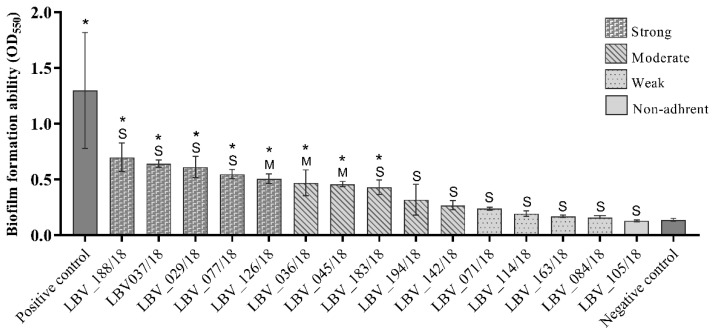
Biofilm formation ability of the *E. coli* strains from pet pyometra. Each column represents the mean of optical density at 550 nm (OD_550_) of the respective *E. coli* strain. The column design represents the biofilm formation ability classified as strong, moderate, weak and non-adherent. The “S” symbolizes strains isolated from uterus histopathology classified as severe pyometra degree. The “M” symbolizes strains isolated from uterus histopathology classified as moderate pyometra degree. *: statistically significant compared to negative control at one-way ANOVA test. *Staphylococcus epidermidis* ATCC 35984 was used as positive control. Wells contained only broth were used as negative control.

After demonstrating that the majority of the samples produced components of the extracellular matrix, we analysed the biofilm formation capacity of the 15 *E. coli* strains isolated from pyometra cases. The results demonstrate that almost all *E. coli* isolates (93.3%) were able to produce biofilm with different adhesion abilities (Table 2). One strain was classified as non-adherent (6.7%), while four strains were weakly adherent (26.7%), five were moderately adherent (33.3%), and five were strongly adherent (33.3%) (Table 2 and Figure 2).

Of the 15* E. coli* strains, 8 strains (53.3%) had significantly higher (*p* < 0.05) optical densities then negative controls (Figure 2). In assessing biofilm formation capacity by the *E. coli* strains according to the characterization of endometrial histopathological changes, no significant relationship (*p* > 0.05) could be detected between these two variables.

### 2.5. Virulence Genotyping of the E. coli Strains Highlights the Pathogenic Capacity of This Pathotype

The 15 strains identified as *E. coli* were molecularly characterized, including the virulence profile. Firstly, the isolates were subjected to multiplex PCR for Clermont phylogenetic classification. Eleven strains were classified as phylogenetic group B2; two strains (LBV_045/18 and LBV_072/18) as *Escherichia* clade I; one as phylogenetic group F (LBV_142/18) and one as unknown (LBV_194/18) (Table 2).

**Figure 3:**
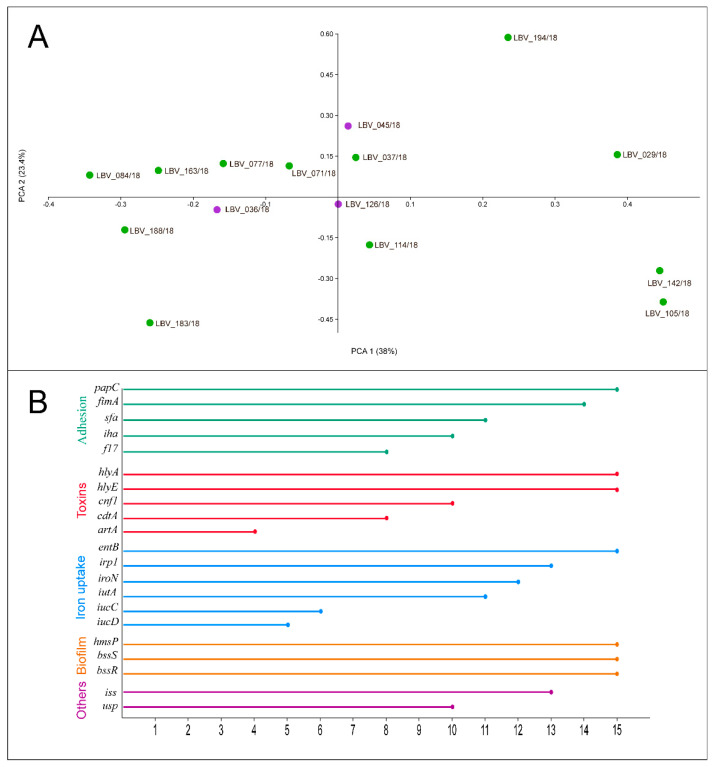
(**A**) 2D Principal Coordinate Analysis (PCoA) visualization of *E. coli* strains from pet pyometra. Data were computed from the Dice’s distance matrix with the virulence determinants. Strains are coloured based on their endometrial histopathology lesion classification: purple: moderate lesion; green: severe lesion. (**B**) Frequency of virulence genes identified by PCR assay in the *E. coli* strains. Each line represents the frequency that the respective virulence gene was detected by PCR method in the 15 *E. coli* strains of this study. The genes were grouped and coloured based on *E. coli* virulence factor classification, related to adhesion, toxicity, iron uptake systems, biofilm formation, and others (bacteriocin—*usp*; invasion—*iss*).

All virulence genes were detected in at least one *E. coli* strain. Genes *hlyE*, *hlyA*, *papC*, *entB*, *bssS*, *bssR*, and *hmsP* (15 isolates, 100%) and *fimA* (in 14 isolates, 93.3%) presented the highest frequency, followed by *irp1* and *iss* both presents in 13 isolates (86.7%); *iroN* (12 isolates, 80%); *iutA* and *sfa* (11 isolates, 73.3%); *iha*, *cnf1*, and *usp* (10 isolates, 66.7%); *cdtA* and *f17* (eight strains, 53.3%); *iucC* (six isolates, 40%); *iucD* (five isolates, 33.3%), and *artA* (four isolates, 26.7%) (Figure 3B).

Finally, the PCoA analysis was performed to identify virulence determinant relationship among the *E. coli* strains and examine the grouping of *E. coli* strains according to endometrial histopathological lesion degrees—moderate or severe lesions (Figure 3A). PC1 had the largest variance, with 36.5% captured by PC1 and 22.6% captured by PC2. A high divergence among the points was observed. Although only three *E. coli* strains were related to moderate histopathological lesion in contrast to 12 strains that were related to severe lesions, no relationship could be identified between the groups.

## 3. Discussion

In this study, we have analysed pet pyometra correlating microbiological and endometrial histopathological aspects. Thirty uterine samples and their respective purulent intrauterine contents were collected from female companion animals with pyometra infection. Bacteriological growth was absent in four purulent intrauterine contents, although the pathological analysis demonstrates different lesion degrees on correspondent uterine tissue samples. This fact could be related to a low number of bacteria on the endometrium in initial cases (in samples classified as mild) and in chronic cases (samples classified as moderate and severe lesions).

Thirty bacteria isolates were recovered from 26 intrauterine content. In this context, *E. coli* was the most prevalent (57.7%) specie. The other bacteria genus isolated (*Bacillus*, *Enterococcus*, *Staphylococcus*, *Streptococcus*, and *Pseudomonas*) had been previously identified in the lowest proportions in pyometra cases [6,7]. Interestingly, *Staphylococcus* spp. was only identified in association with other species in our study (*Bacillus* sp., *E. coli*, or *Streptococcus* sp.). In a recent study, *Staphylococcus* spp. was described as part of the uterus microbiome of healthy bitches [8]. Taking this into consideration, the growth of this genus seems to be proportionate by uterus microbiome imbalance during pyometra infection. 

Since *E. coli* was the most frequent bacteria isolated and correlated to severe endometrial lesions on histopathological analysis (*p* < 0.05) we performed a deeper analysis with these strains. The analysed *E. coli* were classified as Endometrial Pathogenic *E. coli* (EnPEC), according to their genotypic and phylogenetic characterization. In the same way as in this study, the most of EnPEC strains were classified as B2 phylo-group member [4,9,10]. With both uterus tissue samples applied for the IHC technique, new information about the severity of EnPEC pathogenicity on the endometrium was elucidated. The assay provided a visualization of *E. coli* adhesion to the lining epithelium, as could be verified by the strong biofilm capacity production and extracellular matrix production, in addition to the presence of biofilm regulation genes on the *E. coli* strains isolated from the same samples (LBV_029/18 and LBV_037/18), as in most strains of this study.

The high frequency of rdar fimbriae expression and the presence of *fimA* and *papC* adhesion genes on the *E. coli* strains infer that these fimbriae are essential for endometrium infection establishment. A recent study [11] demonstrated that 75% of *E. coli* had significantly higher optical densities than negative controls in a biofilm formation test. These results were higher than the results of our study (44% with the same statistical analysis). Actually, it was expected that biofilm formation capacity on in vitro assay would be lower than in vivo. Biofilm substrate (in vitro condition) do not express adhesins cell receptors (in vivo condition) and the anchorage of bacteria cells to the plate could be deficient, harming the biofilm formation in some strains of this study. Taking this into account, we suppose that future assays performed on in vitro endometrial cells substrate will better characterize the biofilm capacity of EnPEC strains.

In the present study, the IHC technique also clarifies one of the mechanisms used by EnPEC for infection maintenance: cell invasion. The intracellular persistence facilitates bacteria immune and antimicrobial evasion. This mechanism is used by Uropathogenic *E. coli* (UPEC) to invade bladder epithelial [12] and vaginal epithelial cells [13]. Sheldon et al. [14] analysed bovine endometrial cell invasion by EnPEC, demonstrating that invasion represents an important virulence factor for this pathotype in bovine. Although bovine and companion animals’ EnPECs have different phylogenetic aspects [10], we could speculate that, similar to EnPEC from bovines, the EnPECs from canines and felines also promote endometrial cells invasion.

We suppose that the Iss protein can be an important virulence factor associated to cell invasion, given the high frequency of the *iss* gene in the 15 *E. coli* strains of this study, associated to the IHC results of the *E. coli* invasion potential. Given that Iron Acquisition Systems are important in bacteria intracellular maintenance [12], the presence of four different siderophore systems (enterobactin, yersiniabactin, aerobactin, and salmochelin), in the 15 isolates of this study, similar to previously demonstrated [10], suggests that EnPECs have potential to establishment as intracellular reservoir.

The ectatic lesions and endometrial gland necrosis observed in the moderate and severe histopathology degrees are probably produced by bacteria toxins in the addition of inflammatory response (neutrophilic infiltrate). Even though the hemolysins seem to be important to host cells damage, our results of the search for six toxin-related genes (including hemolysins producers) allow us to speculate that a unique hemolysin should not be responsible for endometrial destruction. 

No statistical differences were found on the gene virulence profile between strains related to severe or moderate histopathology degrees, as demonstrated by PcoA analysis (Figure 3). This result infers that all *E. coli* strains of this study have a virulence capacity for severe pyometra establishment. Thus, moderate lesion classification can be dependent on other factors, such as non-gene expression, early pyometra infection stage, and host immunity.

Clinical data, in addition to pathological and microbiological aspects, could classify the pyometra cases better and help one understand pyometra pathogenesis. However, we have no information on vaginal discharge, previous hormonal therapy and the period between estrus manifestation and the first clinical signs of the analysed animals.

In conclusion, our results support the idea that microbiological aspects of pet pyometra not only are important for correct treatment conduct, but also for knowledge about pyometra pathogenesis. The assays performed with the EnPEC strains demonstrate the great genetic and phenotypic adaptation of this *E. coli* pathotype for endometrial colonization. Features such as the adhesion, invasion and destruction of endometrial cells justify the high frequency of *E. coli* associated to severe pyometra cases.

## 4. Materials and Methods

### 4.1. Collection of Samples from Pyometra Cases

The study included 30 females diagnosed with pyometra infection (23 bitches and 7 queens). Intrauterine content samples and uterus fragments were collected during ovariohysterectomy procedure. The females diagnosed with spontaneous pyometra demonstrated lethargy, anorexia and intrauterine liquid content at ultrasound examination after the estrus period. In addition, eight females (five bitches and three queens) submitted to elective spay were included in the analysis (control group), with the collection of uterine secretion, uterus and ovary fragments. Sample collections were approved by the UFRGS Ethical Committee on Animal Use (No. 31874).

### 4.2. Bacterial Isolation 

Intrauterine contents were plated on sheep blood agar (5%) and MacConkey agar. Inoculants were incubated under anaerobiosis, microaerophilia, and aerobiosis atmosphere at 37 °C for 48 h. The microorganisms were identified and characterized according to MacFaddin [15]. The identity of each isolate was confirmed by Matrix Assisted Laser Desorption Ionization Time of Flight (MALDI-TOF) mass spectrometry (Microflex LT instrument, Bruker Daltonik, Bremen, HB, Germany) and MALDI Biotyper 3.1 software (Bruker Daltonik, Bremen, HB, Germany). The analyses were carried out using full protein extraction from pure colonies according to the manufacturer’s instructions, considering a score confidence ≥2.0 to specie-level identification [16,17]. Data were obtained in triplicate for each isolate.

### 4.3. Endometrial Histopathological Analyses

Fragments from the uterine horn and ovary were fixed in 10% neutral buffered formalin. Tissue samples were routinely processed for histopathology and stained with hematoxylin and eosin (H&E) followed by microscopic evaluation. Based on the observed histological lesions, three lesions classification groups were proposed: (i) mild cases: histological alterations restricted to the endometrium; (ii) moderate cases: histological lesions in the myometrium, and (iii) severe cases: mixed and accentuated histological lesions, including necrosis. Ovary analysis was performed to identify the presence or absence of corpus luteum.

Uterine sections from two animals, which had *E. coli* isolated in the purulent content (LBV_029/18 and LBV_037/18), were subjected to immunohistochemistry (IHC) assay using the peroxidase-labelled universal polymer method (MACH 4 Universal HRP-Polymer—Biocare Medical, Pacheco, CA, USA) with a primary polyclonal antibody anti-*E. coli* (ViroStat, Westbrook, ME, USA), as described by De Lorenzo et al. [18].

### 4.4. In Vitro Biofilm Formation Assay with the E. coli Strains

*E. coli* strains isolated from the analysed cases (*n* = 15) were twice subcultured on Luria Bertani agar (LB) without NaCl and incubated aerobically at 37 °C for 24 h. After growing, the colonies were re-suspended in 3 mL of sterile saline solution (0.85%). Bacterial suspension turbidity was adjusted using the McFarland scale 5 (108 UFC/mL) [19]. The qualitative assay for biofilm formation was performed in 96-well sterile plates using bacterial suspension in LB broth. Thus, plates were aerobically incubated at 37 °C for 24 h. *Staphylococcus epidermidis* ATCC 35984 was used as strong biofilm producer control. Wells only containing LB broth were used as a negative control. Each strain was analysed in biological quadruplicate and technical triplicate, being the final classification based in the mean of all analysis.

Biofilm staining was performed according to [19]. The absorbance was measured at 550 nm (OD_550_) in an automated Multiskan FC Microplate Photometer (Thermo Fischer Scientific, MA, USA). Taking into account the absorbance measured in the negative control wells, the strains were classified, as proposed by Stepanović et al. [19], into the following categories: non-biofilm producer (OD_biofilm_ ≤ OD_control_), weak biofilm producer (OD_control_ < OD_biofilm_ ≤ 2 × OD_control_), moderate biofilm producer (2 × OD_control_ < OD_biofilm_ ≤ 4 × OD_control_), and strong biofilm producer (4 × OD_control_ < OD_biofilm_).

### 4.5. Expression of Extracellular Matrix Components in the E. coli Strains

The ability of the *E. coli* strains from this study to express curli and cellulose phenotypic were evaluated according to [20], with some modifications. One colony was seeded as a spot culture on LB agar without NaCl, supplemented with 100 mg/L of Congo Red (Sigma-Aldrich, Saint Louis, MO, USA) and incubated aerobically at 37 °C for 48 h. After growth, the isolates were classified according to their morphology into four groups: rdar (red, dry and rough—curli expression and cellulose production); pdar (pink, dry and rough—cellulose production only); bdar (brown, dry and rough—curli expression only); and saw (smooth and white—no curli and cellulose expression) [21].

Cellulose production was characterized by streaking isolates onto LB plates containing 200 mg/L of calcofluor (fluorescent brightener 28, Sigma-Aldrich, Saint Louis, MO, USA) and incubation at 37 °C for 48 h. Cellulose production was judged by the presence of fluorescence under UV light (366 nm). Isolates that showed fluorescence were considered to be cellulose producers. The phenotypic expression of curli and cellulose was performed in biological and technical duplicate assays.

### 4.6. Virulence Genotype of the E. coli Strains

The genomic DNA of the *E. coli* isolates (*n* = 15) was extracted by the boiling method. Three to four colonies were collected from sheep blood agar (5%), suspended in 50 µL of ultrapure water, chilled on ice for 5 min and then boiled for 10 min at 100 °C followed by centrifugation at 10,000× *g* for 3 min. The supernatant was used as a DNA template in PCR amplifications.

To characterize the virulence profile of the *E. coli* strains, 21 gene markers related to adhesion (*papC*, *fimA*, *f17*, *sfa*, and *iha*), toxins production (*cdtA*, *cnf1*, *hlyE*, *artA*, and *hlyA*), iron uptake system (*irp1*, *entB*, *iutA*, *iucD*, *iucC*, and *iroN*), serum survival (*iss*), bacteriocin action (*usp*), and biofilm regulation (*bssS*, *bssR*, and *hms*P) were selected based on an *E. coli* genome previously analysed [10] (Table S1). The PCR reactions were prepared with GoTaq DNA polymerase (Promega, Madison, WI, USA), following the manufactures instructions. Individual PCR assays were performed to each gene marker according to the description of Lopes et al. [10] (Table S1). The positive control for PCR reactions was the *E. coli*_LBV005/17 strain [10]. The assignment of phylogenetic groups (A, B1, B2, C, D, E, F, and *Escherichia* clade I) amongst the *E. coli* isolates was determined by the multiplex PCR-method (*arpA*, *chuA*, *yjaA*, and *TspE4.C2*), as described by Clermont et al. [22].

The virulence binary matrix was used to explore the relationship of present/absent virulence genes on the *E. coli* strains with endometrial histopathological lesion degrees via multivariate analyses. A Principal Coordinates Analysis (PCoA) was plotted based on a Dice coefficient using a similarity matrix of the present/absent virulence genes with PAST (v.4) [23] and a transformation exponent of c = 2 [23,24]. PCoA allowed one to maximally correlate the distances in the ordination diagram with the linear distance measures in the distance matrix.

### 4.7. Statistical Analysis

The sample size for comparison with two proportions (control group—females without pyometra—and test group—females with pyometra infection) was calculated using Chi-square test by GraphPad StateMate 2.0 (GraphPad Software, La Jolla, CA, USA). The group size was selected assuming 99% of power (true increase 0.80 and *p* < 0.05).

One-way ANOVA followed by Dunnet’s multiple comparisons test (*p* < 0.05) was performed by GraphPad Prism 8.0 (GraphPad Software, La Jolla, CA, USA) to determine statistical differences in the *in silico* biofilm formation assays. The same software was used to assess relationships between uterine severity degrees and in silico biofilm potential of the *E. coli* strains by the non-parametric Chi-square test (X^2^).

PAST (v. 4) software [23] was used to perform a one-way ANOVA followed by Tukey’s test to determine statistical differences among bacterial species isolated from uterus histopathological classified as mild, moderate or severe severity. The Cochran–Armitage test for trends was performed by WinPepi (v. 11.65) [25] to evaluate the correlation among degrees of uterine lesions and the most frequent bacterial specie isolated in the studied cases. 

## Figures and Tables

**Table 1:** Bacterial isolation and degree of endometrial histopathological lesion in each sample from pyometra cases.

Sample ID	Endometrial Severity Degree	Host	Bacterial Isolation *
LBV_024/18	Mild	Canine	*Bacillus* sp. + *Staphylococcus* sp.
LBV_038/18	Mild	Feline	No growth
LBV_041/18	Mild	Canine	No growth
LBV_043/18	Mild	Canine	*Enterococcus* sp.
LBV_036/18	Moderate	Feline	*Escherichia coli*
LBV_045/18	Moderate	Canine	*Escherichia coli*
LBV_054/18	Moderate	Canine	*Streptococcus* sp.
LBV_070/18	Moderate	Feline	*Enterococcus* sp.
LBV_072/18	Moderate	Canine	*Escherichia coli* + *Staphylococcus pseudintermedius*
LBV_115/18	Moderate	Canine	No growth
LBV_121/18	Moderate	Canine	*Streptococcus* sp.
LBV_126/18	Moderate	Feline	*Escherichia coli*
LBV_179/18	Moderate	Canine	*Enterococcus* sp.
LBV_029/18	Severe	Canine	*Escherichia coli*
LBV_034/18	Severe	Feline	*Staphylococcus pseudintermedius* + *Streptococcus* sp.
LBV_037/18	Severe	Canine	*Escherichia coli*
LBV_053/18	Severe	Canine	No growth
LBV_071/18	Severe	Feline	*Escherichia coli*
LBV_077/18	Severe	Canine	*Escherichia coli*
LBV_084/18	Severe	Canine	*Escherichia coli*
LBV_105/18	Severe	Canine	*Escherichia coli*
LBV_112/18	Severe	Canine	*Streptococcus* sp.
LBV_114/18	Severe	Feline	*Escherichia coli*
LBV_133/18	Severe	Canine	*Enterococcus* sp.
LBV_142/18	Severe	Canine	*Escherichia coli*
LBV_163/18	Severe	Canine	*Escherichia coli*
LBV_183/18	Severe	Canine	*Escherichia coli*
LBV_188/18	Severe	Canine	*Escherichia coli*
LBV_189/18	Severe	Canine	*Pseudomonas aeruginosa*
LBV_194/18	Severe	Canine	*Escherichia coli*
LBV213/18	NA	Canine	Negative control
LBV235/18	NA	Canine	Negative control
LBV256/18	NA	Feline	Negative control
LBV262/18	NA	Canine	Negative control
LBV249/18	NA	Canine	Negative control
LBV214/18	NA	Feline	Negative control
LBV240/18	NA	Feline	Negative control
LBV256/18	NA	Feline	Negative control

* Bacterial isolation from purulent content. Identification was performed with MALDI-TOF. NA: no alterations. Negative control: females without pyometra infection signals.

**Table 2:** Overview of the ability from the *E. coli* strains to biofilm formation and phylogenetic groups classification.

*E. coli* Strain	Host	Histopathology Severity Degree	Phylo-Group	Curli Fimbriae	Cellulose Production	Biofilm Formation
LBV_036/18	F	Moderate	B2	rdar	Positive	Moderately
LBV_045/18	C	Moderate	B2	rdar	Positive	Moderately
LBV_126/18	F	Moderate	B2	saw	Negative	Strongly
**LBV_029/18**	C	Severe	B2	rdar	Positive	Strongly
**LBV_037/18**	C	Severe	B2	rdar	Positive	Strongly
LBV_071/18	F	Severe	B2	rdar	Positive	Weakly
LBV_077/18	C	Severe	B2	rdar	Positive	Strongly
LBV_084/18	C	Severe	B2	saw	Negative	Weakly
LBV_114/18	C	Severe	B2	rdar	Positive	Weakly
LBV_183/18	F	Severe	B2	rdar	Positive	Moderately
LBV_188/18	C	Severe	B2	rdar	Positive	Strongly
LBV_163/18	C	Severe	Clade I	rdar	Positive	Weakly
LBV_105/18	C	Severe	Clade I	rdar	Positive	Non-adherent
LBV_194/18	C	Severe	Unknown	saw	Negative	Moderately
LBV_142/18	C	Severe	F	saw	Positive	Moderately

Strains in bold: applied for IHC technique. F: feline; C: canine.

## Supplementary Materials

The following are available online at https://www.mdpi.com/article/10.3390/pathogens10070833/s1, Table S1: Designed primers for virulence genes search in *E. coli* isolates from pet pyometra.
